# Role of chaperones and ATP synthase in DNA gyrase reactivation in *Escherichia coli* stationary-phase cells after nutrient addition

**DOI:** 10.1186/2193-1801-3-656

**Published:** 2014-11-06

**Authors:** Alejandra Gutiérrez-Estrada, Jesús Ramírez-Santos, María del Carmen Gómez-Eichelmann

**Affiliations:** Department of Molecular Biology and Biotechnology, Institute of Biomedical Research, National Autonomous University of México, P.O. Box 70228, México City, 04510 México

**Keywords:** *Escherichia coli*, DNA gyrase, Stationary phase, ATP synthase

## Abstract

*Escherichia coli* stationary-phase (SP) cells contain relaxed DNA molecules and recover DNA supercoiling once nutrients become available. In these cells, the reactivation of DNA gyrase, which is a DNA topoisomerase type IIA enzyme, is responsible for the recovery of DNA supercoiling. The results presented in this study show that DNA gyrase reactivation does not require cellular chaperones or polyphosphate. Glucose addition to SP cells induced a slow recovery of DNA supercoiling, whereas resveratrol, which is an inhibitor of ATP synthase, inhibited the enzyme reactivation. These results suggest that DNA gyrase, which is an ATP-dependent enzyme, remains soluble in SP cells, and that its reactivation occurs primarily due to a rapid increase in the cellular ATP concentration.

## Background

Cellular DNA molecules are under helical tension or negative supercoiling (SC), which is essential for DNA metabolism. DNA topoisomerase enzymes modulate the DNA SC level. In *Escherichia coli*, the level of DNA SC primarily depends on the activities of a type IA DNA topoisomerase I, Top1, and of a type IIA topoisomerase, DNA gyrase. Top1 introduces transient single-stranded breaks and relaxes DNA, whereas DNA gyrase introduces negative supercoils into DNA molecules in an ATP-dependent reaction. DNA gyrase, which is a heterotetramer of two GyrA and two GyrB subunits, introduces a DNA double-strand break, passes a segment of double-stranded DNA (T segment) through the break, and rejoins the DNA, introducing two DNA supercoils per reaction. GyrA introduces the DNA double-strand break, binds covalently to the DNA 5′ends and reseals the break, whereas GyrB has ATPase activity (Champoux 2001; Nöllmann et al. 2007; Chen et al. 2013). The majority of type II DNA topoisomerases (e.g., *E. coli* Top IV and eukaryotic enzymes) hydrolyze ATP to catalyze reactions that do not require energy input, whereas DNA gyrase utilizes the free energy generated by ATP hydrolysis to increase DNA SC, which is an energetically unfavorable process. It has been proposed that ATP also contributes to controlling the separation of the DNA gyrase protein-protein interfaces to prevent the formation of DNA double-strand breaks (Bates et al. 2011). DNA gyrase plays an essential role in resolving the topological changes generated by the DNA unwinding induced by the replication, recombination, repair, and transcription machineries and in separating interlinked replicated DNA molecules during cell division (Nitiss 2009; Chen et al. 2013).

The DNA SC level is regulated by a homeostatic control mechanism that maintains this level within a narrow range to ensure efficient DNA metabolism. Stress conditions that induce DNA relaxation, heat shock (Ogata et al. 1994; Camacho-Carranza et al. 1995; Lara-Ortíz et al. 2012) or starvation (Reyes-Domínguez et al. 2003) require a cellular response to recover adequate SC levels for growth at high temperatures or for cellular growth re-initiation when nutrients are added to the culture. The observed rapid recovery of the SC level during a severe heat shock response occurs primarily due to the disaggregation and reactivation of DNA gyrase by the DnaK-ClpB bichaperone system (Lara-Ortiz et al. 2012) and to an increase in the ATP/ADP ratio (Camacho-Carranza et al. 1995). DNA gyrase reactivation during this response does not require the chaperone GroE; however, this chaperone plays an important role in preventing protein aggregation in growing cells and in cells under stress conditions (Horwich et al. 1993; Gottesman and Hendrickson 2000; Dahiya and Chaudhuri 2014).

Stationary-phase (SP) cells recover SC once nutrients become available. This recovery, which is observed at the beginning of the lag phase, does not require transcription or protein synthesis, is RpoS (σ^38^)-dependent and is inhibited by novobiocin, an inhibitor of GyrB (Reyes-Domínguez et al. 2003). The amounts of GyrA and GyrB proteins in growing and SP cells are similar, whereas the transcription of the *gyr* genes increases approximately 60 min after nutrient addition (Reyes-Domínguez et al. 2003). The delay in the transcription of the *gyr* genes most likely occurs due to the rapid increase in the cellular concentration of the Fis protein, which is a negative regulator of the transcription of the *gyr* genes, observed after nutrient addition (Schneider et al. 1999). These results show that pre-existing DNA gyrase molecules in SP cells are responsible for the rapid recovery of the SC level observed when nutrients are added to the culture (Reyes-Domínguez et al. 2003). This finding suggests that the enzyme is protected from the protein oxidation and aggregation observed in SP cells (Maisonneuve et al. 2008a) or that the enzyme present in aggregates is solubilized and reactivated by chaperones, as in the heat-stress response (Lara-Ortiz et al. 2012). The amount of the main cellular chaperones DnaK and GroE and of the primordial chaperone polyphosphate (polyP) increases in SP cells (Rao and Kornberg 1996; Dukan and Nyström 1998). PolyP is a linear, flexible polymer of inorganic phosphate, Pi, linked by phosphoanhydride high energy bonds present in organisms ranging from bacteria to mammals (Kornberg et al. 1999; Rao et al. 2009). In *E. coli*, the enzyme that synthesizes polyP is the polyphosphate kinase PPK, which catalyzes the transfer of the γ-phosphate of ATP to polyP. This reaction is reversible; however, PPK preferentially synthesizes polyP from ATP (Ahn and Kornberg 1990). PolyP participates in different cellular processes. *E. coli ppk* SP cells exhibit an important decrease in the amount of polyP, reduced RpoS expression, induced SOS genes, sensitivity to oxidative, osmotic and thermal stress and decreased ribosomal translational efficiency and cell viability (Shiba et al. 1997; Kornberg et al. 1999; Tsutsumi et al. 2000; McInerney et al. 2006; Rao et al. 2009). These different phenotypic changes are partially due to a decrease in polyP, which is a primordial chaperone that protects proteins against stress-induced unfolding and aggregation (Gray et al. 2014).

The reactivation of DNA gyrase, which is an ATP-dependent enzyme, in rich media occurs within approximately 30 sec-1 min (Reyes-Domínguez et al. 2003). Transcriptional activation of genes coding for the enzymes that utilize the specific carbon source present in the medium also occurs immediately after adding fresh medium to SP cells (Madar et al. 2013). The rapid reactivation of DNA gyrase is essential for recovering the SC level to allow the transcription of those genes. ATP synthase could contribute to providing the initial ATP molecules required for the reactivation of this enzyme.

In this context, the objective of this study was to evaluate the roles of the main cellular chaperones (e.g., DnaK, ClpB and GroE) and of polyP, which is a primordial chaperone, as well as the roles of glucose and ATP synthase in the observed DNA gyrase reactivation after nutrient addition to SP cells.

## Results

### DNA gyrase reactivation in *rpoH*and *groE*mutant cells in SP after nutrient addition

In exponentially growing *E. coli* cells, a small percentage of total proteins (1–3%) form aggregates (Maisonneuve et al. 2008b). This percentage increases in cells under stress conditions, e.g., heat shock or starvation; under both stresses, the cellular amount of chaperones and proteases also increases (Dukan and Nyström 1998; Maisonneuve et al. 2008a). DNA gyrase molecules aggregated in cells during a heat-shock response are disaggregated and reactivated by the bichaperone system DnaK-ClpB (Lara-Ortiz et al. 2012). DNA gyrase molecules of SP cells recover their activity once nutrients become available. DNA gyrase reactivation, as in heat stress, does not depend on transcription or protein synthesis (Reyes-Domínguez et al. 2003); however, the molecular regulatory mechanism of this recovery remains unclear.

To evaluate the role of the main cellular chaperones on DNA gyrase reactivation after nutrient addition to SP cell cultures, the DNA SC level was studied in strains BB7222 (wt) and BB7224 (Δ*rpoH*). Strain BB7224 lacks the transcription factor RpoH (σ^32^), which regulates the expression of the major cytosolic proteases and chaperones as DnaK and ClpB, and therefore has very low levels of these heat-shock proteins (Kusukawa and Yura 1988; Tomoyasu et al. 2001). This strain carries a second mutation that causes a four-fold increase in the amount of chaperone GroE compared with the wild-type strain; this increase allows Δ*rpoH* cells to grow at 30–37°C (Kusukawa and Yura 1988). The role of chaperone GroE was studied in strains C600 (wt) and CAG9310, which carries the temperature-sensitive mutation *groEL140* (Strauss et al. 1988).

**Figure 1:**
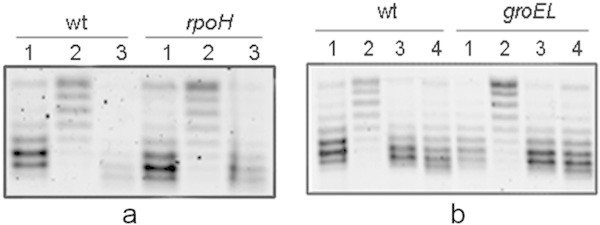
**DNA gyrase reactivation in stationary-phase cells with low levels of the main chaperones.** Cells were grown in LB-MOPS medium at 30°C. Strains used included the following: a) BB7222 (wild type) and BB7224 (Δ*rpoH*), and b) C600 (wild type) and CAG9310 *groEL140* bearing the reporter plasmid pMS01. Strain BB7224 expresses very low levels of the main cellular chaperones, except for GroE, while CAG9310 carries the temperature-sensitive *groEL140* mutation. To induce the recovery of the DNA SC level in stationary-phase cells, cell cultures were diluted in pre-warmed LB-MOPS medium. **a**: 1) Exponentially growing cells, 2) 48 hr stationary-phase cells, and 3) stationary-phase cells diluted 1:10 in LB-MOPS at 30°C and incubated for 5 min. **b**: 1) Exponentially growing cells, 2) 48 hr stationary-phase cells, 3) stationary-phase cells diluted 1:30 in LB-MOPS at 30°C and incubated for 5 min, and 4) stationary-phase cells diluted 1:30 in LB-MOPS at 43°C and incubated for 5 min. Before dilution in LP-MOPS at 43°C, SP cell cultures were incubated for 15 min at 43°C. Plasmid topoisomers were isolated and separated on 1% agarose gels containing 10 μg/mL chloroquine. Migration proceeded from top to bottom. Topoisomers more supercoiled migrated more rapidly in the gel. Similar results were obtained in at least three independent experiments.

The SC level, which is a reporter of DNA gyrase activity, of the pMS01 plasmid was determined in growing and SP cells, as well as in SP cells after nutrient addition. BB7222, BB7224, C600 and CAG9310 cells were grown at 30°C in LB-MOPS medium. To determine DNA gyrase reactivation, wt and *rpoH* SP cells were diluted 1:10 in LB-MOPS medium at 30°C (Figure 1a); on the other hand, wt and *groEL140* cells were diluted 1:30 in LB-MOPS at 30°C and 43°C (Figure 1b). The 1:30 dilution favored a fast temperature increment of the LB-MOPS medium. DNA gyrase reactivation in SP cells grown at 37°C is observed 30 sec-1 min after addition of nutrients (Reyes-Domínguez et al. 2003). This reactivation in wt cells grown at 30°C is observed after 3–5 min.

The results obtained using the Δ*rpoH* and *groEL140* mutants show that the main chaperones involved in preventing protein aggregation and in solubilizing protein aggregates do not participate in reactivating DNA gyrase (Figure 1a and b), suggesting that this enzyme is not present in protein aggregates in SP cells.

### DNA gyrase reactivation in *ppk*mutant cells in SP after nutrient addition

*E. coli* SP *ppk* cells, as mentioned, exhibit an important decrease in the amount of polyP, which is a primordial chaperone that protects proteins against stress-induced unfolding and aggregation (Gray et al. 2014). To determine whether polyP, which increases its amount in SP cells (Kornberg et al. 1999), participates in the rapid reactivation of DNA gyrase after nutrient addition to SP cultures, the DNA SC level was determined in *ppk* mutant cells.

**Figure 2:**
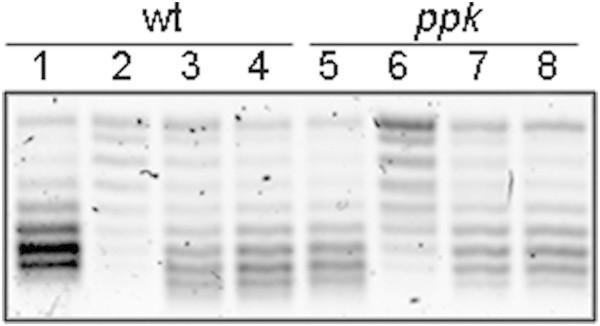
**DNA gyrase reactivation in stationary-phase cells with low levels of polyphosphate, polyP.** Cells grown in LB-MOPS media at 37°C. The strains used were BW25113 (wt) and BW25113 Δ*ppk*, bearing the reporter plasmid pMS01. To induce the recovery of the DNA SC level in stationary-phase cells, cell cultures were diluted 1:10 in pre-warmed LB-MOPS medium. 1) Exponentially growing cells, 2) 48 hr stationary-phase cells, 3) stationary-phase cells diluted 1:10 in LB-MOPS and incubated for 30 sec, and 4) stationary-phase cells diluted 1:10 in LB-MOPS and incubated for 1 min. Plasmid topoisomers were isolated and separated as described in Figure 1. Similar results were obtained in at least three independent experiments.

SP wild-type and *ppk* cells grown in LB-MOPS at 37°C recover the SC level 30 sec-1 min after the addition of fresh medium; this recovery time is the shortest time reported using LB-MOPS medium (Reyes-Domínguez et al. 2003) (Figure 2). The results obtained with the *E. coli* mutant deficient in the enzyme involved in polyP synthesis suggest that this polymer is not involved in the fast reactivation of the DNA gyrase activity in starved cells after nutrient addition.

### DNA gyrase reactivation in cells exposed to glucose or to ATP synthase inhibitors

Results presented show that the main chaperones and polyP, which is a primordial chaperone, do not participate in the DNA gyrase reactivation observed after nutrient addition, suggesting that this reactivation most likely depends on a rapid increase in the cellular ATP concentration.

To explore whether the addition of glucose, which is a carbon source, to SP cells induces DNA gyrase reactivation, SP cells grown in LB-MOPS at 37°C were diluted 1:30 in a 0.86% NaCl solution with or without 0.4% glucose. The 1:30 dilution was used to reduce the number of molecules present in the SP cultures. Nutrient addition to SP cell cultures induces the lag phase. This phase divides into lag1 and lag2. During lag1, no biomass growth occurs, and most of the promoters are inactive, except for those of genes coding for the enzymes that utilize the specific carbon source present in the medium (Madar et al. 2013). During lag2, the biomass increases, and the cell physiology switches to a growth program of promoter activity; however, no cell division occurs. After these two phases, the cell number begins to increase, and after several generations, the cells initiate maximal exponential growth. SP cells grown in rich medium and diluted in rich medium present an extremely short lag1; however, this phase is longer when cells are diluted in minimal medium (Madar et al. 2013).

**Figure 3:**
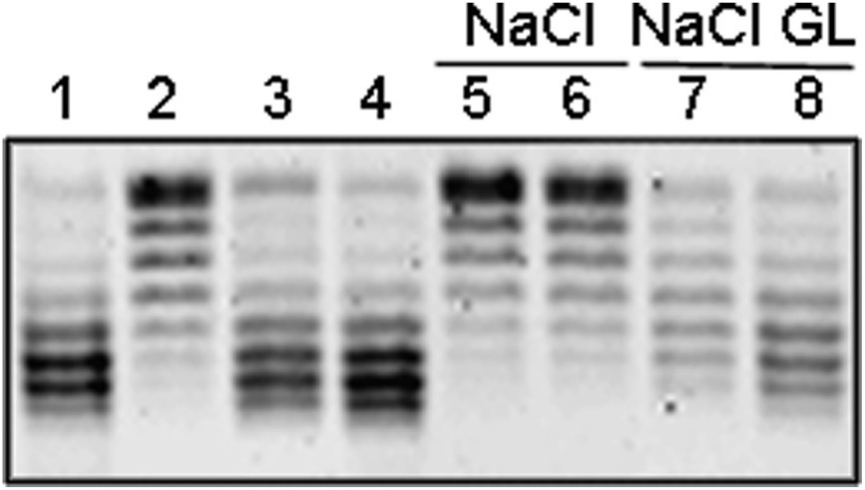
**Effect of glucose as carbon source on DNA gyrase reactivation in MC4100 stationary-phase cells.** Cells were grown in LB-MOPS at 37°C. To induce the recovery of the DNA SC level in stationary-phase cells, cell cultures were diluted 1:10 in pre-warmed LB-MOPS or 1:30 in a 0.86% NaCl solution with or without 0.4% glucose. 1) Exponentially growing cells; 2) 48 hr stationary-phase cells; 3) and 4) stationary-phase cells diluted in LB-MOPS and incubated for 3 or 5 min, respectively; 5) and 6) stationary-phase cells diluted in 0.86% NaCl and incubated for 3 or 5 min, respectively; 7) and 8) stationary-phase cells diluted in 0.86% NaCl-0.4% glucose and incubated for 3 or 5 min, respectively. Plasmid topoisomers were isolated and separated as described in Figure 1. Similar results were obtained in at least three independent experiments.

As shown in Figure 3, SP cells recover DNA SC 3 min after dilution in LB-MOPS, whereas plasmid DNA remains relaxed in cells 3 or 5 min after dilution in 0.86% NaCl. However, cells diluted in 0.86% NaCl and 0.4% glucose partially recover the DNA SC level after 3 min of incubation and completely recover after 5 min. A plausible explanation is that after SP cell dilution in NaCl-glucose, the activation of genes specific for glucose, which is the added carbon source, induces energy production, e.g., ATP.

Another approach to study the participation of ATP molecules in DNA gyrase reactivation involved using ATP synthase inhibitors: Resveratrol (RVT), piceatannol (PCT), and sodium azide. Polyphenols, such as RVT, PCT and quercetin, inhibit *E. coli* ATP synthase. RVT and PCT inhibit ATP synthesis and ATP hydrolysis, whereas quercetin inhibits only ATP hydrolysis (Dadi et al. 2009). The inhibitory effects of these polyphenols were demonstrated by *in vitro* assays using purified F_1_ or F_1_F_0_ membrane preparations from *E. coli* cells grown in LB medium. RSV and PCT, but not quercetin, inhibit cell growth in liquid LB medium. The growth yield in the presence of these two polyphenols was 44% (Dadi et al. 2009). ATPase activity in *in vitro* experiments and cell growth in LB cultures were inhibited when the cells were exposed to 400 μM RVT or 100 μM PCT (Dadi et al. 2009). In *E. coli* cells, RVT and PCT most likely prevent the synthetic and hydrolytic activity of ATP synthase by blocking the clockwise and anti-clockwise rotation of the γ-subunit of the enzyme, respectively, similar to the mechanism observed in bovine mitochondria (Gledhill et al. 2007). Sodium azide is a potent inhibitor of ATP synthase which inhibits ATP hydrolysis, but not ATP synthesis (Gledhill et al. 2007; Zharova and Vinogradov 2004).

**Figure 4:**
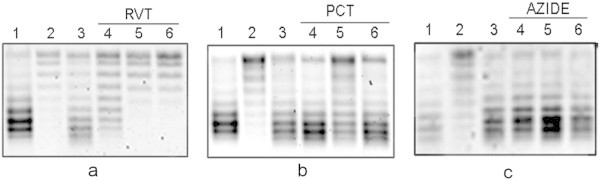
**Effect of ATPase inhibitors resveratrol (RVT), piceatannol (PCT) or sodium azide on DNA gyrase reactivation in MC4100 stationary-phase cells.** Cells were grown at 37°C in LB-MOPS medium. To induce the recovery of the DNA SC level, cell cultures were diluted 1:10 in pre-warmed LB-MOPS medium with or without the inhibitor. **a**: 1) Exponentially growing cells, 2) 48 hr stationary-phase cells, 3) stationary-phase cells diluted in LB-MOPS media and incubated 1 min, 4), 5) and 6) stationary-cells diluted in LB-MOPS with RVT 400 μM, 1.2 mM, or 2.0 mM, respectively. The diluted cultures were incubated 1 min. **b**: 1) Exponentially growing cells, 2) 48 hr stationary-phase cells, 3) stationary-phase cells diluted in LB-MOPS and incubated 1 min, 4), 5) and 6) stationary-phase cells diluted in LB-MOPS with PCT 100 μM, 200 μM or 300 μM, respectively. The diluted cultures were incubated 1 min. **c**: 1) Exponentially growing cells, 2) 48 hr stationary-phase cells, 3) stationary-phase cells diluted in LB-MOPS and incubated 10 min, 4) stationary-phase cells diluted in LB-MOPS-sodium azide 3 mM and incubated 10 min, 5) stationary-phase cells diluted in LB-MOPS-sodium azide 5 mM and incubated 5 min, 6) stationary-phase cells diluted in LB-MOPS-sodium azide 5 mM and incubated 10 min. Plasmid topoisomers were isolated and separated as described in Figure 1. Similar results were obtained in at least three independent experiments.

To determine the effect of RVT, PCT, and sodium azide on the recovery of DNA gyrase activity, the DNA SC level was determined in SP cells grown in LB-MOPS and in SP cells diluted 1:10 in LB-MOPS with or without the ATP synthase inhibitor. DMSO or DMSO-RVT, PCT or sodium azide added 1 hr before diluting the SP cells, did not modify the plasmid topoisomer distribution in these cells. The recovery of the DNA SC level was complete after 1 min incubation of SP cells diluted in LB-MOPS; whereas the recovery of SP cells diluted in LB-MOPS with 400 μM RVT was partial, this recovery was completely inhibited by 1.2 mM RVT and 2.0 mM RVT(Figure 4a). PCT 100 μM induces an inhibitory effect similar to 400 μM RVT *in vitro* or in cell cultures (Dadi et al. 2009). However, 100 μM, 300 μM or 500 μM PCT did not inhibit DNA SC recovery (Figure 4b). The effects of RVT and of PCT in cell cultures were determined after 20 hr of growth in LB medium (Dadi et al. 2009), whereas, in this study, the effect on DNA SC recovery was determined 1 min after polyphenol addition to SP cells. The different inhibition levels observed with RVT and with PCT most likely occur due to higher cell membrane permeability for RVT than for PCT. The RVT molecule has three hydroxyl groups compared with four in PCT. Sodium azide (1 and 3 mM), which is a potent inhibitor of ATP synthase which inhibits ATP hydrolysis, but not ATP synthesis (Gledhill et al. 2007; Zharova and Vinogradov 2004), did not inhibit DNA gyrase recovery (Figure 4c).

The effect of glucose and the inhibitory effect of ATP synthase inhibitors on DNA gyrase activity, which were detected by the recovery of the DNA SC level, strongly suggest that an increase in ATP molecules in the first minutes of lag1 plays an important role in this recovery.

## Discussion

Studies on the initial regulatory events occurring after nutrient addition to SP cells, which are in the lag1 phase, are relatively few. A recent study (Madar et al. 2013) reported that no biomass growth occurs during lag1; however, promoters of genes coding for enzymes that utilize the carbon source present in the fresh medium added to SP cells are selectively activated. Previously, we demonstrated that the reactivation of DNA gyrase molecules present in SP cells occurs 30 sec-1 min after the addition of fresh LB-MOPS medium (Reyes-Domínguez et al. 2003). This reactivation, as shown in this study, is inhibited by the ATP synthase inhibitor RVT. In addition, RpoS (σ^38^) a regulator of SP genes, is degraded in the first minutes after addition of glucose to SP cultures. This degradation, essential to favor the transcription by RpoD (σ^70^) of genes needed to re-initiate cell growth, is controlled by an increase in ATP levels (Peterson et al. 2012).

The amount of intracellular ATP in mid-log phase *E. coli* cells is 1–5 mM, depending on the cell growth conditions and methodology used, this amount decreases two- to threefold in stationary-phase cells (Bochner and Ames 1982; Buckstein et al. 2008) and rapidly increases during lag1 (Murray et al. 2003). However, DNA gyrase activity depends, not on the ATP level but on the [ATP/ADP] ratio. An increase in the ATP/ADP ratio strongly correlates with the increase in the plasmid SC reached *in vitro* with purified DNA gyrase (Westerhoff et al. 1988) or *in vivo* using cells grown in MOPS buffered minimal medium with succinate or glucose as a carbon and free-energy source (Van Workum et al. 1996). The increase in this ratio also correlates with the increase in the plasmid SC observed in the transition of cells to anaerobic growth (Hsieh et al. 1991a) or during a salt stress (Hsieh et al. 1991b). Changes in the [ATP/ADP] ratio in cells grown in LB-MOPS medium or in other media along the lag1 have not been studied.

The present study shows that DNA gyrase reactivation does not depend on cellular chaperones, as has been reported for the heat-shock response (Camacho-Carranza et al. 1995; Lara-Ortiz et al. 2012), or on polyP, which is a primordial chaperone (Gray et al. 2014) and a potential energy source (Kornberg et al. 1999). Glucose, which was the sole nutrient added to SP cells in this study, favored this reactivation, whereas RVT, which is an inhibitor of ATP synthase, induced complete inhibition. The phenotype of the strains BB7224 Δ*rpoH*, and CAG9310 Δ*ppk* used in this work suggests, at most, a partial redundancy of the functions displayed by the main chaperones and primordial chaperone polyP. As mentioned before, strain BB7224 Δ*rpoH* shows very low levels of chaperones, but it carries a second mutation that causes an increase in the amount of chaperone GroE. This increase allows BB7224 cells to grow at 30–37°C. The Δ*rpoH* strain lacking this second mutation, grows only at very low temperatures (Kusukawa and Yura 1988). The CAG9310 Δ*ppk* cells, which express all the main chaperones, show in SP an important decrease in the amount of polyP, and among other phenotypic changes, the sensitivity to oxidative, osmotic and thermal stress increases and a cell viability decreases (Kornberg et al. 1999; Rao et al. 2009). The double mutant BB7224 Δ*rpoH* Δ*ppk* could help to explore in log and stationary-phase cells, the possibility of main chaperones/polyP redundant functions.

During the SP, the stop codon read-through and shifting of the translation reading frame (accuracy of the ribosomes) decrease, generating an increase in unfolded proteins, which are prone to aggregate (Barak et al. 1996; Wenthzel et al. 1998). The amount of DNA gyrase in SP cells is similar to that of exponential cells (Reyes-Domínguez et al. 2003), suggesting that no translation of the *gyr* genes occurs during the SP. This finding partially explains why DNA gyrase reactivation after the SP does not depend on cellular chaperones in contrast to DNA gyrase reactivation during the heat shock response.

In summary, our previous results and the results presented in this study strongly suggest that an increase in the [ATP/ADP] ratio occurs during the first seconds of lag1. This increase reactivates the DNA gyrase molecules present in the SP cells before the addition of fresh medium and induction of lag1, leading to a rapid recovery of the DNA SC level.

Future work regarding the lag1 phase must focus on the timing of these events, on the relative cellular concentrations of ATP and ADP in SP cells grown in different media and conditions, as well as on the role of RpoS (σ^38^) in DNA gyrase reactivation.

## Conclusions

A plausible regulatory mechanism operating during lag1 to prepare cells to enter lag2 begins with an increase in the ATP/ADP ratio required for DNA gyrase reactivation and RpoS degradation. Fast DNA gyrase reactivation is essential to seal potentially dangerous DNA double-strand breaks (Bates et al. 2011), to recover the DNA SC level (Reyes-Domínguez et al. 2003), and together with RpoS proteolysis (Peterson et al. 2012), to activate the promoters of rRNA genes (Murray et al. 2003), and of genes which code for enzymes that use the specific carbon source present in the medium (Madar et al. 2013).

## Methods

### Bacterial strains and growth conditions

The bacterial strains used in this study included the following: a) MC4100 *araD139* Δ(*argF*-*lac*)*U169 rpsL150 relA1 flbB5310 deoC1 ptsF25 rbsR* (Tomoyasu et al. 2001); b) BB7222 as MC4100 but *araD*^+^ and BB7224 as BB7222 but Δ*rpoH*::Km (Tomoyasu et al. 2001); c) C600 *thi thr leu tonA supE lacY*-*lacZX*-*90* (Strauss et al. 1988); d) CAG9310, C600 but *groEL140 zjd*::Tn*10* (Straus et al. 1988); e) BW25113 *rrnB3 lacZ4787 hsdR514* Δ(*araBAD*)*567* Δ(*rhaBAD*)*568 rph-1* (Baba et al. 2006); and f) BW25113 Δ*ppk* (Baba et al. 2006; Coli Genetic Stock Center CGSC, Keio Collection). Nutrient-rich Luria-Bertani (LB) medium containing 40 mM morpholinopropanesulfonic acid (LB-MOPS, pH 7.4) was used throughout this study. This medium was used to avoid cell exposure to alkaline stress due to the high pH reached by the SP cultures in LB medium. Cultures (10 ml) in 125 ml Erlenmeyer flasks were grown in LB-MOPS medium at 30°C or 37°C with shaking (180 rpm) to reach the SP and then incubated 48 hrs.

### DNA supercoiling analysis

The DNA SC levels in exponentially growing cells, SP cells, and SP cells diluted in fresh medium were determined using the pMS01 plasmid, which is a tetracycline-sensitive derivative of pBR322 (León et al. 1988), as a reporter. This plasmid avoids SC-level interferences due to expression of the membrane protein TetA (Lodge et al. 1989) and to transcription of the two divergent promoters present in pBR322. The distribution of plasmid topoisomers was obtained as described elsewhere (Camacho-Carranza et al. 1995). Briefly, before plasmid DNA isolation, cultures were immediately chilled by transferring them to tubes containing LB-MOPS crushed ice. A fast decrease in temperature is needed to inhibit enzymatic activities that can modify the level of plasmid SC. Plasmid DNA was isolated by the clear alkaline lysate method (Sambrook et al. 1989), and plasmid topoisomers were separated by electrophoresis in 1% agarose gels containing 10 mg/ml chloroquine. At this chloroquine concentration, the topoisomers that were more supercoiled before electrophoresis migrated more rapidly in the gel. The electrophoresis was run in Tris-borate-EDTA buffer (Sambrook et al. 1989) at 25 V for 22 hr.

## Authors’ information

G-E A, obtained her Master in Science with a thesis related with her study on DNA gyrase reactivation in stationary-cells after addition of nutrients; R-SJ is an academic technician member of the Department of Molecular Biology and Biotechnology; G-E MC is head of Laboratory, and member of the Department of Molecular Biology and Biotechnology and of the SNI (National System of Researchers, México).
